# Women’s health at work: a qualitative study on women’s health issues in relation to work participation. Experiences and perspectives from female teachers and managers in Norwegian high schools

**DOI:** 10.1186/s12889-024-19241-y

**Published:** 2024-06-30

**Authors:** Marianne Gjellestad, Heidi Enehaug, Kristin Haraldstad, Vegard Nilsen, Migle Helmersen

**Affiliations:** 1 Department of Nutrition and Public Health, Faculty of Health and Sport Sciences, University of Agder, Kristiansand, 4604 Norway; 2 Work Research Institute, Centre for Welfare and Labour Research, Oslo Metropolitan University, Oslo, 0176 Norway; 3 Department of Health and Nursing, Faculty of Health and Sport Sciences, University of Agder, Kristiansand, 4604 Norway; 4 Department of Public Health, Agder County Municipality, Arendal, 4809 Norway

**Keywords:** Occupational health, Public health, Female employees, Working life, Sick leave, Recognition

## Abstract

**Background:**

Public health and working life are closely related. Even though Norway is one of the world’s most equality-oriented countries, working life is still divided by gender. Women have a lower rate of participation in working life than men, they work more part-time and they have a higher sickness absence. Research has mostly focused on structural and cultural reasons for gender differences, rather than on the fact that women and men have different biology and face different health challenges. The aim of this project was to explore experienced associations between women’s health and female participation in working life.

**Methods:**

Qualitative methods were chosen for investigating women’s experiences. We carried out in-depth interviews with 11 female high school teachers and supplemented the material with a focus group with five managers from the same organisation. The interviews were recorded and transcribed verbatim. We used the six steps of reflexive thematic analysis for consistency in the analysis process.

**Results:**

The teachers shared a variety of experienced health issues within the field of women’s health and perceived barriers in the work environment. Four main themes were identified: (1) invisibility of women’s health at work, (2) complexity and lack of recognition of women’s health at work, (3) women’s health in work environment and (4) women’s health and role conflicts. There were few contradictions between the two informant groups. We found that health, work and total life intertwine and that complexity, lack of recognition and invisibility of women’s health appear at different levels in a mutual influence: for the women themselves, in the organisation and in society.

**Conclusion:**

Lack of recognition and invisibility of women’s health in the work environment is suggested to influence women’s work participation. The complexity of female health is not captured by gender-neutral structures in the work environment meant to protect and promote employees’ occupational health. Recognition of women’s health in the work context can therefore contribute to a gender-equal, health-promoting and sustainable working life.

## Background

Health and work are closely related. Work participation is in itself health promoting, both on an individual level and for public health, and a healthy workforce is needed to maintain services in the welfare state [1, 2]. Women constitute half of the potential workforce, and improvement of public health and gender equality is addressed in the context of working life specifically in two of the United Nations Development Goals: Gender Equality (5) and Decent Work and Economic Growth (8) [3].

### Work and work environment

A substantial work and welfare system known as The Nordic Model defines Norway’s approach to the occupational setting [4]. Pillars of this model are tripartite cooperation on work environment structures and welfare programmes and the inclusion of persons with reduced work ability in the workforce, despite their health challenges [4, 5]. Norway is one of the world’s most equality-oriented countries, but working life is still divided by gender. Women work more part-time compared with men (37.3% vs. 16.7%), and they have almost double the rates of sickness absence (7.1% vs. 4.1%) [6–9]. Until recently, national and international research has mostly focused on structural and cultural causes for lower female participation and women’s higher rates of sickness absence. Women’s double roles, which consist of combining paid work, domestic work and other responsibilities, have been studied from different angles without achieving a common understanding for the gender difference in sickness absence [7, 10–13] as have gender differences in attitudes and norms [14, 15].

Systematic work with health, safety and environment (HSE) has historically been developed for industrial, traditionally male-dominated workplaces, focusing on the physical environment and security [16]. More recently, typical challenges and demands in female-dominated occupations have been included in the HSE systems, such as violence and threats, role conflicts, and relational and emotional demands [16–18]. A perceived balance between demands and resources in the work environment is related to work ability, job satisfaction and coping [19–21]. High relational and emotional demands are associated with a higher risk for exhaustion and burnout and are thus important factors for higher sickness absence in women [22–24]. Work–home role conflicts are also found to impact health negatively, with a larger impact for women than for men [13, 25].

### Women’s health

In addition to structural and cultural gender differences in working life, women and men also have difference in biology and experience different health challenges. In Norway, the biological and physiological differences between women and men have previously been suggested to potentially impact work participation and sickness absence [11, 18], but the implications of women’s specific health issues have been poorly investigated in this regard. Women’s health can be defined as

diseases and ailments that only women have, diseases that affect more women than men, that affect very many women or that have different consequences for women than for men [26].

Historically, medical and health research has largely been conducted on men and considered ‘gender neutral’, with the consequence of not taking female biology into consideration. A result of this is that we still know less about women’s health in general and how it impacts the work context [26].

The menstrual cycle and the female life stages entail physiological and psychosomatic variations and transitions, which are normal processes; however, they involve ailments and diseases that only affect women. Recent international research has shown how female ailments can affect work ability and sickness absence in women [27], such as in pre-menstrual syndrome (PMS) [28, 29], dysmenorrhea [30], endometriosis [31] and menopause [32]. Pregnant employees’ work environment, work ability and sickness absence have been addressed in national and international studies over the last 25 years [33–35]. While many workplaces today implement facilitation for their pregnant employees, pregnancy is still identified as the primary reason for the higher rate of sickness absence among women in Norway, accounting for 20% of the gender gap [36]. International studies have also shown that infertility treatments may negatively affect work ability and sickness absence [37, 38]. Female employees returning to work following maternity leave have the legal right to take breaks for breastfeeding. However, a national report on workplace discrimination suggests that enforcing this right can be problematic [39], a finding corroborated by international research [40, 41]. Furthermore, diseases that predominantly affect women are often chronic and complex, such as conditions involving pain or fatigue [7]. These conditions, along with hormonal and reproductive issues, make the examination of female employees’ experiences in the workplace particularly pertinent.

### Study rationale

International research has shown that women’s health issues can negatively affect work participation, work ability and sickness absence, but more knowledge is needed both on the specific conditions and the complexity of women’s health in the context of work [26, 42]. The objective of this study is to deepen the understanding of how women experience associations between women’s health issues and work participation, and further, to explore how different perspectives from women and their managers reveal different challenges related to women’s health at work.

Norwegian high school teachers represent a profession in which, despite being employed on equal terms, women have lower participation through more part-time work and higher rates of sickness absence compared with men [43]. As such, they constitute a relevant sample for this study. Two research questions were formulated:

RQ 1: How do female teachers experience women’s health in relation to work participation?

RQ 2: How do female teachers and high school managers experience potential challenges for work participation related to women’s health?

## Methods

### Study design

A qualitative, inductive approach was chosen to explore the informants’ experiences. The data material consisted of in-depth interviews with 11 female teachers, supplemented by a focus group with managers from the same organisation. Designed within the framework of social constructionism, the project benefitted from the diverse background of the research team, which encompassed women’s health, occupational and social medicine, human resources (HR), HSE and sociology. This diverse expertise enhanced our understanding and reflexivity. Situated at the intersection of women’s health, working life and public health, the project drew on theories and research from all three disciplines. For the analysis we used reflexive thematic analysis by Braun & Clarke [44, 45].

### Data collection

Informants were recruited from high schools in a region in the southern part of Norway. Recruitment of female teachers was done through the unions’ representatives. The representatives sent a mail with information about the study to all their members, and so teachers interested in participating were encouraged to directly contact the first author. Information was also posted openly and to all on the schools’ website. Also here, teachers were encouraged to contact the first author if they wanted to participate. Consequently, 11 women were recruited for in-depth interviews.

All the teachers were parents, although some of their children were already adults and no longer living at home. Table 1 provides further details about the teachers’ background characteristics.

**Table 1:** Characteristics of the teacher informants

Teachers	Age	Teacher seniority (years)	Living situation	Children living at home
Teacher 1	41–55	10+	Partner/spouse	Yes
Teacher 2	25–40	2–10	Partner/spouse	Yes
Teacher 3	25–40	2–10	Partner/spouse	Yes
Teacher 4	56–67	10+	Partner/spouse	No
Teacher 5	25–40	2–10	Partner/spouse	Yes
Teacher 6	41–55	10+	Partner/spouse	Yes
Teacher 7	56–67	10+	Single resident	No
Teacher 8	56–67	10+	Partner/spouse	Yes
Teacher 9	41–55	10+	Partner/spouse	Yes
Teacher 10	41–55	10+	Partner/spouse	Yes
Teacher 11	41–55	10+	Partner/spouse	Yes

The interviews were semi-structured. An interview guide was developed based on areas of women’s health and aspects of women’s life expected to influence work participation, such as family situation, work environments, stress and coping [6, 17, 46]. The first question in the interview guide was ‘how do you understand the term women’s health?’, a question intended to open for personal reflections and experiences. The first author conducted the interviews, and each interview lasted for 60–90 min. Locations used were private meeting rooms at the work places and facilities at public places, depending on the wish of the informant.

Recruitment of managers to a focus group was done through the organisation’s HR department. Information was distributed by e-mail to all managers, with an open invitation to participate, and managers interested were encouraged to directly contact the first author. Five managers signed up. See Table 2 for background characteristics of the informants. The managers worked at different levels in the organisation, and certain details were left out of the informant characteristics to ensure anonymity. Their span of control varied with direct personnel responsibility from six to 28 persons, and they were all responsible for both female and male employees.

**Table 2:** Manager informant characteristics

Managers	Male/female	Manager seniority (years)	Manager for general studies/ vocational studies
Manager 1	F	10+	Both
Manager 2	F	0–2	General studies
Manager 3	F	10+	Both
Manager 4	F	10+	Both
Manager 5	M	0–2	Vocational studies

The focus group interview took place at a county house and lasted for 120 min, facilitated by the first and last authors together. The interview guide used for the teachers’ interviews was adopted and used as a starting point for the conversation.

All interviews were conducted over the period of April 2022–April 2023. Both the in-depth interviews and the focus group were recorded and transcribed verbatim by the first author.

### Analysis

The interviews with teachers were analysed first, as the study’s main material. The six steps of reflexive thematic analysis [44, 45] were used to ensure a systematic analysis process. In the first step, familiarisation of the data, the material was read and key terms were identified. In step two, we created 300 codes based on identified meaning units in the text. The codes were merged into 234 and divided into groups with subgroups. Coded material was analysed, and four main themes were identified based on the research questions as step three. In step four, the themes were examined and renewed in relation to the whole dataset before themes and subthemes were formulated in step five. Coding and analysis were data-driven and done by the first author, with discussion with the author team for each step. NVivo software was used for the structure and overview of the material. Examples of the analysis process from raw data to themes are given in Table 3.

**Table 3:** Examples of the analysis: process from raw data to coding and themes

Quotation	Meaning unit/coding	Sub-theme	Main theme
“I was so young; I didn’t dare to leave work. But I couldn’t sleep, and I was so angry…” (Teacher 4)	Pre-menstrual syndrome (PMS)	Areas of invisible women’s health	Invisibility of women’s health at work
“I was told not to share too much about the reason why I had to go. It was better just to say it was health-related, because of the shame.” (Teacher 2)	Women’s health issues are unaddressed and attached to shame	Lack of recognition of women’s health	Complexity and lack of recognition of women’s health at work
“Now, we have forced through that two of the restrooms are separated for men and women. And for teachers, only for teachers.” (Teacher 3)	Separate rest rooms for women	Women’s health and physical work environment	Women’s health in work environment
“It’s like a sauce. And the sleeping problems… it really affects my health. The nights I have slept well, the weeks I am well rested, I feel like a different person…” (Teacher 11)	Complex situation and sleep problem affects health	Invisible women’s health in work‒home conflicts	Women’s health and role conflicts

The material from the focus group with managers was also analysed according to the three first steps of reflexive thematic analysis [44], but from step four the themes were adapted to themes and sub-themes developed from the teachers’ material, in order to reveal variations or contradictions within the two informant perspectives. Information from all informants was included in the analysis, although not all were quoted in the final report.

### Ethical considerations

Participation was voluntary for all informants, and written informed consent was obtained. To address any potential emotional distress due to recalling sensitive experiences during the interviews, informants could, if necessary, contact the organisation’s occupational health service post-interview. The informants were anonymised during transcription and identified only by numbers. Quotations in the results are specified as to informant group and number. All transcribed material was securely stored in a password-protected user area on the university server. The project received ethical approval from the Faculty of Health and Sport Sciences at the University of Agder and the SIKT- Norwegian Agency for Shared Services in Education and Research (SIKT ref.798,255). The study was designed and conducted in accordance with the principles of the World Medical Association (WMA) Declaration of Helsinki [47].

## Results

Four main themes were identified from teachers and managers’ shared experiences: (1) invisibility of women’s health at work, (2) complexity and lack of recognition of women’s health at work, (3) women’s health in work environment and (4) women’s health and role conflicts. (Table 4).

**Table 4:** Themes and sub-themes from the analysis

Themes	Sub-themes
Invisibility of women’s health at work	Areas of invisible women’s health: Pre-menstrual syndrome, menstruation, menopause, infertility, pregnancy, breastfeeding, chronic conditions due to female biology
Complexity and lack of recognition of women’s health at work	Complexity of women’s health
	Lack of recognition of women’s health
Women’s health in work environment	Women’s health and physical work environment
	Women’s health and psychosocial work environment
	Women’s health and organisational work environment
Women’s health and role conflicts	Invisible women’s health in work–home conflicts
	Additional tasks disproportionally assigned to women can impact health

An initial finding from the teachers’ interviews was a lack of difference between the informants who defined themselves as ‘healthy’ at recruitment and those who reported having issues regarding women’s health. Several of the ‘healthy’ informants initially talked about their good health, but as the interviews proceeded, they shared stories that revealed a variety of health challenges. Together, the 11 teachers had personal experiences with a wide range of female conditions at work. They all had experiences with menstruation, one had been through infertility treatment, all had children and five had reached the age for potential menopausal symptoms. One suffered from lymphoedema, one from polycystic ovary syndrome (PCOS) and four suffered from chronic pain due to migraine or musculoskeletal disorders. Seven had also experienced exhaustion or burnout throughout their careers. When asked about the term women’s health, the teachers described a context of complexity, in association with general health, responsibility for children, family, household, work and the total life situation.

### Invisibility of women’s health at work

#### Areas of invisible women’s health

The teachers described how different female conditions were invisible and disregarded in the work context. Menstruation was considered something normal and something private, even though it was described how PMS and dysmenorrhea affected health and work:

You had no excuse because it was not a disease. It’s like being pregnant, it’s not a disease. So just work on. And the symptoms… well, they may feel like a disease. And they had been, if the reason was different. (Teacher 2)

I was on the pill because I had to regulate it… so I always had my period on a Thursday. Then I could go to work on Friday and stay home Saturday and Sunday. Those days I had to stick to the house, because I couldn’t even go to the mailbox without bleeding through my pants. I just couldn’t be out among people. I had to plan everything. Of course, it affected quality of life and everything. (Teacher 7)

Menopause was hardly addressed in the workplace, according to the teachers. They described shame and stigma and how it felt unnatural or inappropriate to talk with their manager about it, especially if the manager was a man. The symptoms could be challenging:

You are observed all the time when in the classroom. It has been uncomfortable with the hot flushes and all, but you cannot do much about it. Menopause has been difficult. (Teacher 9)

The invisibility of women’s health issues in the workplace was confirmed by the managers; there was no general focus on the topic in the work environment and little openness around the specific conditions.

When working while pregnant, the teachers expected facilitation from their employers. However, for several of them, lack of facilitation resulted in sickness absences. The flexibility in the teachers’ work situation should make it possible for them to regulate the level of activity themselves and take breaks when they needed, some of them had been told by the management. One was followed up by a midwife coming to talk with her and her manager together, according to a local routine. Her experience was that the employer used this to disclaim responsibility, as no changes were made in her work situation:

It was only that talk, and then ‘Now you must try to stay at work’. But it is a shared responsibility, as I see it. Of course, I want to stay at work. But they must make it possible. (Teacher 5)

Breastfeeding at the work place was a negative experience for most of the teachers interviewed. Some, who had tried to get time off to continue breastfeeding when returning to work after maternity leave, described how the employer gave flexibility – but did not reduce the workload – and so they had to make up for the lost time later, sometimes at home after working hours. The managers agreed that time off to breastfeed was difficult to sort out in practice, but they claimed this was mainly because the guidelines were unclear and the teachers’ expectations unrealistic. Teachers and managers both described difficulties with planning for breastfeeding at work because the babies’ feeding schedule was hard to predict.

Several of the teachers suffered from chronic diseases affecting their work ability and periodically resulting in sickness absence. One, who had both lymphoedema and fibromyalgia, described how she used the rest of the day after work to heal to be able to work full-time. Since lymphedema was partly unrecognised as a medical diagnosis, she also felt responsible for managing the situation alone. She wanted to save money for an operation to relieve the pain but struggled to work full-time with reduced work ability because of the symptoms.

#### Complexity and lack of recognition of women’s health at work

Women’s health was described as a complex and poorly recognised field by both teachers and managers, and they claimed that it was not taken into consideration when women’s participation or the higher sickness absence was discussed in the workplace. Several of the teachers described how women’s health issues tended to be diagnosed as psychological conditions by general practitioners, especially if the symptoms were complex and diffuse. One informant explained how she experienced the complexity:

I think, regarding the diseases that mainly frame women, it is a bit unclear what it is all about. Some of the diagnoses are looked down upon, you know, like ‘women’s diseases’. What women get, because they take on too much, like it’s their own fault. The difference is that the diagnoses are so diffuse that it is difficult to know what comes from the disease and what comes from the rest of the situation. So it might be that if I only pull myself together… then it will be okay. (Teacher 7)

The managers mainly put the blame for the higher sickness absence on women’s double burden. However, the managers also defined women’s health in terms of its complexity and stated there was a lack of knowledge on women’s health and potential consequences for work participation.

It’s easy to think that women’s health is only about menstruation, reproduction and menopause, the obvious things. But I believe there are other differences between men and women as well. And if you look at the research, all the research done on men’s health. Heart diseases, for instance. And this is about our bodies. It is not about being women in a gender perspective. (Manager 5)

The teachers addressed a lack of knowledge on women’s health, both among themselves and the employers and in society in general. Some described how they were divided between being open about their ailments to reduce stigma and taboos, but at the same time, protecting themselves from discrimination. One of them said:

I do not tell my manager, unsolicited (…). I find it hard to be honest with him because I do not know the consequences… We need more knowledge on this, to make more flexible arrangements in the working life. There’s a difference between equality and equity. It is something about our different bodies. Men and women have different biology. But you are not allowed to say that. We are supposed to be equal, equalised (…). But the fact is that we are equal, but we sometimes have some constraints. At least I have, with my female body (…). And then, if you talk about it, it is difficult to avoid stigma. Because then you are not feministic enough or do not fight for women… or you are simply weak. This is maybe the tricky part with women’s health. (Teacher 2)

The same teacher used her family history to share her view on the status of women’s health in society and working life:

Historically, my generation, we are the first women who are 100% workers and 100% mothers… My grandmother, she had the same disease as me, and stayed at home. My mother, also the same disease, she was home earlier and is now partly on welfare (…). This is not a new situation. It is not like all women now suddenly have got issues, but they were just not employed earlier, so no one knew! This is maybe what provokes me the most, that this is regarded as a new problem, and stigmatised, when they ask why women have more sickness absence… If they had done research on women’s health when my grandmother stayed home, then… we might had known by now. And we might have figured out systems to simply make it easier for women to stay in the workforce. (Teacher 2)

### Women’s health in work environment

Teachers and managers described how work environment had impact on the occupational health of the female employees. The teacher’s role was described as a rewarding position, varied and meaningful, and with great pride and extensive autonomy. Autonomy was important not only for coping with the everyday job situation, but also for staying in the profession over time. Several of the teachers had backgrounds that allowed them to apply for other positions if they wanted to leave their current work situation. However, the teachers described a comprehensive workload and a busy day, handling teaching in the classroom, preparations, administration and care for pupils and their parents. They described challenges in the work environments, both regarding physical, psychosocial and organisational work environment, how the challenges affected their female health and different strategies for coping with the situation.

#### Women’s health and physical work environment

Several of the teachers had experienced how the school’s physical work environment was not adapted for women. One said:

This school was new in 2012, and they still haven’t managed to make wardrobes or restrooms for teachers (…). When I was pregnant, I had to go all the time. Then I had to stand in line with the pupils in the breaks. For men, it may not be so important, but for women, who have their period or are pregnant and must go all the time, it is… yeah. Then you really should have a private restroom. (Teacher 3)

Some of the teachers reported a lack of suitable furniture for those with reduced physical health, particularly for the pregnant staff. One described how she had to use a bar stool when teaching during her first pregnancy, resulting in too much standing and back pain. The managers also expressed the need for female teachers to access customised furniture when pregnant and a wardrobe and restroom of their own:

A separate restroom and wardrobe (…), really, this is not a question of facilitation. This should be a matter of course. (Manager 4)

#### Women’s health and psychosocial work environment

The psychosocial work environment was crucial for coping with the professional role, according to the teachers. Some worked in teams and described the importance of the team’s collegial support, safety and social life. Both teachers and managers claimed that health in general was insufficiently addressed in relation to psychosocial work environment; the HSE work mainly focused on safety rounds and physical conditions, and health was not an issue in the routine performance review. Some of the teachers said that they missed information about preventive measures from the occupational health service or in other instances. In particular, ailments within the context of women’s health were invisible and not addressed in the work environments. As one said:

We do not talk about it (…). If someone comes with plaster or crutches, then we ask what has happened, and we get the story. But never for the invisible conditions, like headache or menstrual pain. (Teacher 6)

Maintaining open communication with managers was deemed crucial for managing reduced work capacity and securing the necessary support. However, due to confidentiality laws regarding diagnoses, addressing specific needs in these discussions could prove challenging. The management was referred to as distant by the teachers, both because they had their office geographically placed far away and too many employees in their span of control. This distance was described as both negative and positive by the teachers; they expected to get less support from their managers, but the distance gave them more personal and professional freedom. However, distance could result in a lack of trust in both directions, which was described as a potential barrier for dialogue if problems arouse. Another challenge in the teachers’ psychosocial work environment was meeting the pupils’ needs and performing in the classroom every day; one described it as ‘wearing a mask’ (Teacher 5). They explained how working with the pupils also made them bring their work home with them in the afternoon after school: mentally, because they needed time to process experiences themselves, but also practically, when pupils or parents called or sent e-mails outside work hours. In addition to this, there was often a need to prepare the next day’s teaching in the evening. The teacher’s role was not considered to be different for men and women in the first place, but several informants described gender differences in their ways of coping with the challenges. Some, both among teachers and managers, claimed that women were more susceptible to relational conflicts and emotional stress, whereas others claimed this was more due to different personalities.

#### Women’s health and organisational work environment

Most of the teachers interviewed experienced the workload as heavy. Several also described a burdensome responsibility that was intrinsic to the position because it was difficult to distinguish between work and leisure time. One said:

Being a teacher is really demanding, because you feel the weight of the job on your shoulders 24 − 7, from August to June. (Teacher 11)

Stress could be both positive and negative, according to all the teachers in our study. But even though the positive stress provided self-efficacy and energy, several said it was sometimes difficult to notice when it turned into negative pressure; at some point, it became difficult to cope with, and for some of them it had resulted in sickness absence. The managers talked about coping as a culture, more than as an individual ability:

For a manager it is all about structures and about developing an organisational culture that recognises the employees. It doesn’t always have to be the manager who gives the recognition (…), it might be a colleague. We strive for a culture of coping and empowerment, where you recognise each other. (Manager 3)

Several of the teachers interviewed had worked with reduced capacity over time because of health problems due to the female biology. They struggled to find a balance for coping with stress and reduced capacity at the same time. Some described how their work capacity to some extent varied with different work tasks and how general well-being at the workplace made it easier to go to work if they did not feel well. When asked about short-term absence, they shared stories on how they were expected to plan for the substitute teacher from home despite being away from work. This made it easier to just go to work anyway if they could. For being able to work despite health problems in the long run, predictability – but at the same time flexibility – in the work situation was pointed out as important factors:

I am very lucky to have a flexible work day. The days I am in too much pain, I can adjust the teaching to make it work (…). I am very lucky to be a teacher. This would be different in other typical female professions, like in health care, for instance. (Teacher 2)

Having part-time work because of health was potentially problematic for different reasons, according to the teachers. Part-time could make it harder to cope with the tasks because you missed the routine, and you lost some of the social and professional network, they claimed. However, reduced health could force you to reduce your position. Several of the teachers described how it was hard to accept this limitation, even though they understood that a reduction was necessary:

In that period when I reduced my position, I felt I did a much better job. I could relax, and then I did not stress like I did before, with just working more and more (…). Now, I could listen to the pupils, instead of only instructing them what to do. So, then I started to realise, maybe the answer is not just to work longer hours. It doesn’t make it better. (Teacher 7)

It would be better to work 70%, or something like that… But it is very stigmatised to receive disability benefits because of women’s health issues. No, that would not be a solution. It would be a better idea to try something else, something less demanding. Especially if we are supposed to work until 67 or 70. I don’t believe it is realistic that teachers or nurses stay in their regular position until they reach that age. (Teacher 8)

Administrative tasks were a special source of stress, according to the teachers, as these came on top of teaching and preparing for classes. The managers had an understanding of this, but explained how new demands came down as an order from the school’s administration. Older teachers explained how experience helped in coping, and they saw the need for extended mentoring programmes for their younger colleagues. It was also about not putting too hard demands on yourself, they claimed. Both informant groups described a difference between male and female teachers in this regard; it was suggested that some women were more vulnerable to stress because they tended to be more critical of themselves. The teachers shared different views and different ways of coping with the workload, as two of them explained:

Many of my female colleagues, they are so extremely critical towards themselves. And often dissatisfied with themselves. They might think ‘Oh, I should have managed that situation differently’, while my male colleagues… Either they do not see how the situation was not optimal, or they just leave it behind. (Teacher 8)

‘Good enough’ is a great philosophy. When I was young, I needed to be 100% sure of the choices I made. Now, I am down to 51%, then I just go for it. It is good enough. This is how I get things done, and how I manage to stay in my position. (Teacher 9)

### Women’s health and role conflicts

All but one of the teachers interviewed held the family’s main responsibility for the children and household. Both teachers and managers claimed that many women had a more family-oriented focus when they had small children, and some argued that this could affect motivation for work and lower the threshold for calling in sick. Both groups expressed concern about how high demands and pressure on full-time working mothers could negatively influence their health. Flexibility in the teaching profession often implies working at home in the afternoon or evening. This was viewed as an asset, but also a burden, as it could increase the role conflicts between work and home.

#### Invisible women’s health in work–home conflicts

The teachers described how role conflicts between work and family life resulted in bad conscience for both, and how this was worsened by the expectations and attitudes they perceived from the society. The balancing of roles affected both quality of life and health, and for some of them, it caused recurrent periods of sickness absence. They explained how they got exhausted from work and then their chronic conditions resulted in less energy to cope with exhaustion, especially in combination with stress. The complexity of the situation made it difficult to identify and sort out health problems from the rest. Invisible and unacknowledged illnesses or conditions were described as particularly problematic in terms of coping, since they resulted in reduced work capacity without the surroundings noticing or acknowledging them. Two of the teachers described how invisible ailments affected their work ability in different ways throughout their careers:

It has been a struggle. When I look back now, I have spent too much energy on the job, to the cost of my family. I believe it has left a lasting impression on my children, who are now grown-ups. They never say it out loud, but I believe they reckon I should have tried harder, because it was not possible for them to see how hard I had already tried. (Teacher 7)

You just ignored it (…) with pain and all… However, I cannot remember that it affected my results. I just had to work harder for my achievements. It’s not fair. (Teacher 8)

Several described how a mix of role conflicts and health problems had made them reduce their position voluntarily to handle the double burden. One explained:

We justified it with care for children and the family situation, because according to the headmaster, this was much easier than to use documentation from the GP (general practitioner) to reduce the position. She said that reduction because of health was much more complicated, so I trusted that was true. I have small children, so then we just did it that way. (Teacher 11)

The teachers also described work capacity and work ability as assets; work was something important to them. Some described how being engaged and productive resulted in energy to fulfil the roles of both mother and teacher. One talked about her friends who received welfare benefits and questioned their choices:

Among seven friends, now only three of us are working. The rest are on welfare for different reasons. But they manage to do a lot, and they keep the house perfect… What I think about it? I believe that many, many more could have done some paid work. I really mean that, but it is difficult to talk about. (Teacher 9)

Both teachers and managers addressed the importance of gender equality when discussing women’s work participation and work ability. Some pointed to the difference in women and men’s reproductive roles and argued how it inevitably affected female participation in working life. Parts of the reproductive burden could not be equalised, as one teacher claimed:

What kind of pressure is now put on women? All of this ‘being equal’… It is good, we need to work on gender equality, if you look at top jobs and leading positions. But there is also a difference between us. Until the day men can produce new life, we just cannot be the same. (Teacher 3)

#### Additional tasks disproportionally assigned to women can impact health

Several of the teachers described how work–home role conflicts worsened when additional tasks were added to the family life, a problem also acknowledged by the managers. In respect to taking care of older parents or other relatives, the teachers described how they felt about the expectations to help and assist. Some shared how they needed to set boundaries to protect themselves and to take care of their own health. One, who had reduced her position because of a chronic condition, said:

I kept it secret from my parents which day I had off work. Otherwise, they had a long list of tasks they needed help for that very day. I need that one day a week to breathe (…). I don’t see people that day. I need to have it quiet. (Teacher 9)

Some of the teachers had children with chronic diseases, and they explained how this, in addition to working a full-time position, made them vulnerable and exhausted. One said:

I have four children, and one of them has functional disabilities (…). I am convinced that if I had support for my daughter, I could have worked full-time. Or, if I could reduce my position to take care of her situation, I would not end up on sick leave. (Teacher 8)

Teacher 8 shared her story on how she continued the logistics of her everyday life because she needed to work full-time to gain rights for a retirement pension. When she was eventually on sick leave, she was offered a cognitive course on stress management, which provoked her:

There should be a system for this. Especially in Norway, where there is a concern about having more children, then there should be more support when children are born with a lower level of functioning. Here something is really missing (…). You are pushed to work, to earn your rights in the society. And then there is no consideration given the fact that women often have these additional burdens. (Teacher 8)

## Discussion

### How does women’s health matter?

While exploring the term ‘women’s health’ in the teachers’ work environment, we found that women’s health issues were unacknowledged and invisible in the context, according to both teachers’ and managers’ experiences. They also explained how this affected both work ability and their resilience to manage work–home role conflicts. Based on these findings, we suggest that lack of recognition of women’s health can appear as both a cause and an effect of the situation. In this section, we elaborate and discuss the implications and consequences of this suggested dynamic.

Staying healthy at work requires a balance between demands and resources [48]. The ability to cope with maintaining this balance is an individual experience; however, the work environment is a structural responsibility regulated by laws [6, 49]. Work shall be health promoting [50, 51], this requires a work situation that is experienced as comprehensible, manageable and meaningful [21].

### Complexity and invisibility – the lack of recognition of women’s health

Our informants defined the term women’s health as a totality of health, family, work and overall life situation. Both teachers and managers described complexity, lack of knowledge and lack of recognition of women’s health as obstacles to identifying barriers regarding health and work. The ailments were invisible; hormone-related ailments such as menstruation and menopause were considered private matters rather than legitimate reasons for reduced work ability, and chronic conditions were often ignored. We found that both lack of knowledge and lack of recognition were present at different levels according to our informants’ experiences. The individual level, with women themselves keeping the ailments private and unaddressed, the organisational level, with little or no inclusion of women’s health in HSE systems or organisational policies, and finally a societal level, where women were expected to manage their conditions on their own, with all three influencing each other. In terms of coping at work, previous research has shown how the imbalance of demands and resources affects the experience of exhaustion and (dis)engagement [19]. In the short term, demands in the work environment are a given. Resources, however, are affected by support and personal coping strategies. Bakker and Demerouti [52] argue how personal resources matter in this balance and, in turn, predict the job resources. As a result, we argue, the uncertainty that follows working with unrecognised health issues may influence resources and hence the ability to cope with the work environment. This is exemplified by the hidden ailments experienced by the teachers, and not least by those describing how they were torn between being open or keeping their ailments invisible at work, the latter to protect themselves from stigma, shame or potential career consequences. This experience of ambiguity can add strain to the work situation, as the gap itself can cause stress and insecurity. We suggest that the coping strategy of keeping the health conditions invisible can be seen as ‘invisibility work’: the effort of camouflaging unacknowledged health issues due to shame or stigma or to avoid being considered weak.

Women, who make up nearly half of the workforce in Norway, are potentially burdened with hidden constraints from invisible female health issues. This raises the question of why this is not taken into consideration in a modern working life that emphasises inclusion as a main principle. Historically, biological and physiological gender differences have been used as arguments to keep women out of work, educational and societal arenas, as women were regarded as inferior to men [53]. After women increasingly entered the work scene from the 1950s onward, the female body has not been taken into consideration in this context. The work on gender equality has focused on the ideal of women and men being able to do the same tasks and, thus, having the same rights, without exploring potential biological differences. A result of this is that we have less knowledge of women’s preferences and needs in the work context [26].

### Women’s health at work: equal or with equal rights?

The teachers in our study described how they encountered different challenges at work because of their female ailments. Physical, psychosocial and organisational work environments are ‘gender neutral’, with man as the norm [16]. As systems and structures for HSE were originally designed based on the male-dominated work places, laws and regulations have also developed according to this [16, 18]. Over the last decade, rights for pregnant and breastfeeding employees have been included in the Nordic welfare model and in the Norwegian Work Environment Act [49]; nevertheless, our study clearly shows examples of how challenges persist when legislations are not turned into local practices. Other female health issues, such as menstruation or menopause, were not addressed either in laws or policies, or by the women themselves, according to our informants. Hence, if resulting in reduced work ability or sickness absence, they remained individual limitations for the women and concealed problems for the responsible managers. The importance of implementing women’s health in local or organisational policies for more equal participation has been suggested in international research [32]. Physical limitations in working environments, such as not having adequate accessibility to facilities, are found to be challenging for women in conditions such as pregnancy [54], breastfeeding [40], menstruation [55] and menopause [32, 56]. We argue that ensuring equal rights for women and men in this case requires different treatments, which means organising the physical work environment in a way that will allow women to participate on equal terms. The physical work environment has been proven to be a fundamental element for job performance and potential exhaustion [48], providing a basis for balancing demands and resources. The fact that teachers and managers presented similar views regarding the physical work environment likely indicates that the actual conditions are the result of history and a lack of consciousness more than of current contradictions between employers and employees.

The risk of being exhausted and overwhelmed by emotional and relational demands in the work environment was also found to be highly relevant for the teachers in our study. Constantly performing in the classroom and meeting pupils and parents’ needs require high levels of emotional and relational work. This type of strain has been suggested as a main cause of women’s high sickness absence in typical female-dominated work places [22, 24]. If women, at the same time, are suffering from invisible or unrecognised health conditions, this may produce stress and ambiguity and, thus, possibly increase the strain, according to our analysis. The complexity described by our informants and the difficulty of sorting out the health issues from the rest of the situation are especially relevant to investigate in relation to emotional work. Emotional work often has an invisible character itself, is difficult to measure [22] and drains more personal resources than a physical strain would do. This can lead to a negative synergy with invisible health problems and reduced resources for coping with the overall situation.

The balance of demands and resources in the work environment predicts outcomes on both the individual and organisational levels; individual experiences occur in a collective context, and the structures guide the organisational outcomes [48]. The systematic work with HSE should reflect the real challenges, and ensuring a fully responsible work environment is an organisational responsibility [49]. However, we argue that not recognising women’s health as a common concern makes the work environment less customised for women than for men, and consequently, women and their managers are left to seek individual solutions to common structural problems.

### *‘*Organising’ life, work and health

Both informant groups presented work–home role conflicts as part of the picture when talking about work participation. The issue of women combining work with the main responsibility for children and the household has been studied from different angles and in different contexts, referred to as ‘the second shift’ [6, 13]. Even though men take more of a part in housework and participate more as caretakers than previously, statistics show that women are still more likely to assume the main responsibility for tasks, planning and organising family life, both in Norway and in other European countries [6, 25]. Women also report more balancing of roles and experiencing role conflicts, and previous research has found that women multitask more than men, for instance, looking after children, while at the same time, cooking dinner or cleaning the house, whereas men more often report either taking care of children, making food or cleaning [13]. Studies report that work–life balance has an impact on self-reported health, with a higher association between a poor work–life balance and experiencing negative health outcomes found for women than for men [25]. A suggested cause for the higher female association between work–life balance and negative self-reported health is the combination of societal expectations of gender equality and structures and behavioural norms maintaining traditional roles [25].

In keeping with the findings of previous studies on work–life balance and work‒home conflicts, the findings from our study show how complexity, lack of recognition and invisibility of women’s health can complicate the balance and the role conflicts for women additionally. The teachers interviewed experienced extensive freedom and individual flexibility in their work situations. Based on our analysis, this seemed to be two-sided: they could adapt some of the work to their personal schedule, but the freedom also implied a personal responsibility for managing the workload on their own. Our informants described how this meant working at home in the evenings and pushing the limits between work and private life. Women are more at risk of having chronic conditions than men [7], and if working with chronic conditions and reduced work ability, you may be forced to bring work home because you cannot finish the tasks at work within the given time. As for teachers, with the flexible work organisation, a problem of reduced capacity then may not be addressed because they have the possibility of finishing the tasks later. Several of our informants also claimed that women tend to put higher demands on themselves, but it was debated among both informant groups whether this was typical for females in general or more dependent on different personality types, regardless of gender. However, some of the teachers described how they tried to solve the complex situations by just working more hours, until they reached a point where they could not do so anymore due to their health issues. A missing dialogue with the manager also made them vulnerable if the problems reached a crisis point: the managers’ large span of control, with responsibility for many employees, made the individual follow-up more distant, which made a dialogue on taboo topics even less likely. The managers were concerned about structures and about growing a culture of mastery, based on the employers’ experiences. This suggests the possibility of putting new, currently invisible topics on the agenda when developing organisational policies, if this is suggested by the employees. However, the group of managers considered work–home conflicts as the main reason for the female employees’ lower participation, reduced work ability and higher sickness absence. The teachers, on their hand, described how they had been on sick leave – or even reduced their positions, temporarily or permanently – due to the combination of health and work–home conflicts. These were complex situations, according to the teachers, in which the actual health problems were insufficiently addressed or acknowledged.

As women’s health problems are more often chronic and complex than those of men [7, 26], and, as we found in this study, are often unacknowledged or invisible in the work environment, we see a reason to ask whether women have a greater risk of having hidden health problems also in work–home role conflicts. Our data show clear examples of how health and total life situation can intertwine; role conflicts can burden health, and reduced health can drain the resources needed to handle the role conflicts, which, in turn, burdens health and so on.

Our informants claimed that women’s physiological burden of reproductive health was insufficiently acknowledged in work–home role conflicts. They explained how their part in pregnancy, maternity and breastfeeding was expected to run its course ‘as normality’ alongside working full time, except during the defined period of maternity leave. Several described how this made them exhausted and vulnerable. Based on this, we question whether the societal focus on equal parenting has a potential downside of underestimating the reproductive role of women when work and family policies are formed. In Norway, the unpaid extension of public maternity leave has shown a tremendous increase over the last years, with 48% of mothers prolonging the leave from work unpaid in 2021 [57]. Work–home role conflicts and the desire to continue breastfeeding are the most frequently reported reasons for this choice [39, 57].

Reduced work ability is identified as a cause of part-time work by choice [6]. In Norway, the voluntary reduction of positions without a medical certification leads to loss of income and other rights. Inclusion in public welfare programmes requires that each individual work to earn full pension benefits, regardless of care tasks or other obligations. Both teachers and managers in our study described how women were responsible for extra tasks in their family life and how this could lead to reduced health and sickness absence because of overload. Here, the factor of invisible or unrecognised health issues are suggested to add an additional challenge and might put the woman at risk of losing or reducing either health or entitlements to welfare schemes. This can make her more vulnerable and challenged from a gender equality perspective, a factor that has a further impact on health promotion.

Working life is a primary arena for improving public health and equalising social inequalities. Based on our findings, we argue that lack of recognition of women’s health in this context can create and maintain work environments poorly customised for women. The World Health Organization (WHO) has emphasised the importance of working life’s importance for social determinants of health and claims that addressing gender biases in the structures of society is crucial for improving equality and designing fair welfare systems [50]. These arguments are also raised in more recent reports [51] and in action plans for the UN’s Sustainability Development Goals [3].

### Implications for practice – female health in the nordic model

We argue that the time has come to recognise the female physiology and women’s health in the occupational health context on the way towards gender justice and equality in working life. Women’s health represents other potential limitations and barriers in the work environment compared with the health of men, and this should be raised from individual problems to a collective, structural concern to increase inclusion and prevent withdrawal. The aim for female work participation has been explained and justified by national and international plans and goals [3, 5, 49], but the possibilities depend on sociocultural determinants as well as political structures in different countries. In Norway, the inclusive approach, comprehensive working life programmes and welfare systems should include equal rights to ensure a health-promoting and sustainable working life. Based on the results from this study, we suggest a greater focus on female occupational health and inclusion of the life stages of women in existing HSE systems. The established tripartite cooperation on national and local levels serves as a solid foundation for the development of new knowledge-based practices in a modern working life [5].

### Methodological considerations

The design of this study approached women’s health from a broad perspective; hence, it did not provide new knowledge on how specific health conditions influence work participation. However, the qualitative methods gave us in-depth knowledge on experiences important to understanding the needs and challenges for women regarding the work environment. We also found that the type of work and working environment can impact perceptions of health. This strengthens our findings but affects limitations for transferability to other different professions.

The sample of 11 teachers supplemented by a group of five managers was a limited selection, but we consider the data to have sufficient information power [58]. At recruitment, we asked the teachers whether they considered themselves ‘healthy’ or ‘challenged’ in terms of women’s health, and we included half of the informants from each group to ensure diversity in our material. However, an initial finding in our analysis was that most of the teachers had subjective ailments or complaints related to their female health, regardless of whether they described themselves as ‘healthy’ or ‘challenged’ in terms of women’s health when they signed up for interview. For a qualitative study according to subjectively defined health and diagnoses, or analyses of potential variation between a ‘healthy’ or ‘challenged’ group in the context of work, this distinction should be defined as a purposeful inclusion criterion, and the number of informants accordingly increased to make two larger groups of informants to achieve sufficient information power. Since this was not the case for this study, we handled the 11 teachers as one group for the further analysis.

Coping at work was considered important for both teachers and managers interviewed. We acknowledge that coping and self-efficacy at work hold a multitude of factors which is outside the scope of this study and is recommended for later research [21]. Stigma and shame were also concepts addressed by the informants and are identified as relevant for investigations in relation to women’s health in further research.

## Conclusion

Based on our findings, we suggest that complexity, lack of recognition and invisibility of women’s health can influence women’s participation in working life. We found the lack of recognition present at different levels, for the women themselves, in the organisation and in society. The gender-neutral HSE systems of today were originally designed and adopted for men, and the complexity of female health is not captured by structures in the work environment meant to protect and promote the employees’ occupational health. We found that women keep their issues private and unacknowledged in the work environment; this can influence their ability to cope at work and their resilience in work–home role conflicts, which again can maintain practices of keeping women’s health invisible and unrecognised. The two informant groups in our study, teachers and managers, largely shared similar views on women’s health and barriers in the work context. They both also described how they experienced health, work and total life situation intertwining. Women are expected to work full-time, have many children and still take the main responsibility in the home. We argue that to improve gender equality in working life, women’s work environment and occupational health must be recognised and considered based on knowledge of female physiology. This will benefit individuals, organisations and society and lead to a health-promoting and sustainable working life.

## Data Availability

Data generated and analysed in this study are not publicly available due to confidentiality of the informants and ethical restrictions.
